# Compound Dislocation of the Shoulder

**Published:** 1853-07

**Authors:** Eli Hurd


ART. V.—*Compound Dislocation of the Shoulder.* By ELI HURD, M. D. Read before the Niagara Co. Med. Society, and its publication requested by the society.

*Wednesday, Feb.* 18, 1852. Was called to see George W. Timerman, of Shelby, Orleans Co., N. Y., aged thirty years. He had previously enjoyed tolerable good health, was of sanguine temperament, and rather slender constitution.

When I arrived found him sitting in a chair, pale, cold, and shivering, with the left arm resting upon pillows. Pulse scarcely perceptible at the wrist, and the fingers of that arm benumbed and motionless. From the flattening of the deltoid muscle, the friend supposed the arm was broken, and pointed to the spot of fracture. But on examination I found the head of the humerus dislocated downward, and protruding through the integuments in the axilla.

Prompt measures were taken to reduce the luxation. Extension was made from the wrists of either arm, and the assistants directed to pull steadily. The head of the bare and protruding humerus was then seized with thumb and fingers of my left hand, my right resting upon the back and top of the shoulder. The time from the commencement of extension to the completion of reduction, did not exceed thirty seconds—the extensive laceration of the capsular ligament and integuments rendering it comparatively easy. The forearm was then flexed upon the arm and carried across the chest, where it was suspended in a × sling. Sensation, warmth, and prickling, soon returned in the limb, and he was able to move his fingers again. The subclavian vein was lacerated, and on the removal of pressure upon it by the return of the head of the humerus to the glenoid cavity, copious hemorrhage ensued. This was arrested by compression. The wound in the integuments, between three and four inches in length, next received attention. Three sutures were introduced and the water dressing applied. The forearm was then flexed upon arm, and carried across the chest, where it was suspended in a sling. He was now laid on a mattress, in bed, an anodyne given, and perfect rest enjoined. Very severe pain in the abdomen now occurred, which was relieved by a repetition of the anodyne.

This rare accident was occasioned by being unfortunately caught in the gearing of a thrashing machine, and being drawn with great force and violence against it. Several cuts and scratches were visible on the face, but no other marks or contusions were perceptible.

*Feb.* 19, 9, A. M. Patient has passed a restless night. Reaction returned.

6 o'clock, P. M. Shoulder swollen and painful; headache; tongue coated; thirst.

*Feb.* 20. Bowels constipated; complains of pain and soreness in the "cords of his neck," more than in his shoulder; high fever. Ordered a cathartic of sal Epsom, to be followed by alkaline drinks. Continue the water dressing.

*Feb.* 21. Cathartic operated briskly; had perspired freely; much relieved; wound looks well; walks some about his room; arm in sling.

*Feb.* 22. Removed the sutures; wound united by the first intention; some headache; pain in "cords of the neck;" shoulders same as before; tongue coated; fever; thirst. Gave an occasional anodyne; continued the alkaline drinks, and water-dressing.

*Feb.* 24. Elbow hot, painful and swollen; some officious neighbor, since my last visit, had persuaded him to discontinue the cold applications, and substitute whisky and strong camphorated spirit. Corrected this mal-practice; repeated the saline cathartic; continued the other medicines.

*Feb.* 26. Patient much better; fever abated; tongue moist; pain, heat, and swelling less; elbow easier; sits up, and walks about the house. There is no discharge from the wound, nor has there been from the first dressing; skin about the shoulder somewhat discolored; slight fever at night, and restless; straightens the arm nearly, and moves the shoulder, assisted by the other hand.

*Feb.* 29. Patient very much improved; free from fever; shoulder and elbow scarcely swollen; neither painful; "cords of the neck" somewhat sore yet discontinued the water dressing; dress with the ceratum cetacei; trifling discharge from the inner extremity of the wound, near the tendon of the pectoralis major; is cheerful, and rests well. Without permission has been eating pork and beans! corrected this mal-practice also.

*March* 5. Good improvement since last visit; the shoulder joint can be moved with more freedom, and the motion is attended with much less pain.

*March* 11. Patient convalescent; dismissed. The arm, by his own assistance, is moved backward and forward freely; and outward from the body to an elevation of thirty-five degrees, without causing pain.

*April* 10. Called at my office; can clench the fingers; flex the hand and forearm to considerable extent; deltoid muscle paralyzed; not seen since. There was not to exceed two tea-spoonfulls of pus discharged during the treatment.

In the following harvest, he was able to cut his grain and hay; and since, works his arm, I understand, as usual.

